# Dr. Paget on Congenital Malformations of the Heart

**Published:** 1832-01-01

**Authors:** 


An Inaugural Dissertation on Congenital Malformations of the Heart. By *John Paget*, M.D. Royal 8vo. pp. 54. Edinburgh, 1831.

It was a wise regulation of the Senatus Academicus of Edinburgh, that left the option of the language to the writer of a medical thesis. Nothing could be more absurd—nothing more disadvantageous to young graduates, than a compulsion to publish their theses in a language which few of their friends could understand—and which very few indeed of their brethren would take the trouble to peruse. Dr. Paget need not, therefore, make an apology "for neglecting a custom, which has only its antiquity to urge in its behalf."

The subject of congenital malformation has engaged more attention on the Continent than in this country, where our anatomists have generally contented themselves with descriptions of single monstrosities, without relation to any of the important doctrines of physiology, with which they are closely connected. On the Continent they have been more successful, and have elucidated many of the functions of sound and natural structure by imperfections and extravagances, if we may so say, in our organization.

The thesis is divided into three portions; the *first* exhibiting a classification of malformations—the *second* shews the particular malformations themselves—and the *third* delineates the pathological effects, the symptoms, and the treatment. Over the first part we must pass entirely, and the second part can only be cursorily noticed.

The author commences with malformations of the heart. The ductus arteriosus sometimes remains pervious—as does the foramen ovale. Total absence of the heart (acardia) is a very rare occurrence, and is generally found only in conjunction with absence of the brain, or the upper half of the body. Imperfection in the septum ventriculorum is of pretty frequent occurrence, as is the origin of the aorta from both ventricles. The existence of only one auricle, when the ventricles were double, has been noticed by Haller and others. The valves of the heart and of the great vessels, are subject to malformations and imperfections—more especially the sigmoid valves of the pulmonary artery.

In external form, the heart is rarely much altered, except from chronic disease. Absence of the pericardium is extremely rare, but positively proved by Breschet. We must pass over the malformations of position, which are numerous, and very puzzling as to their cause. Such are the chief congenital malformations hitherto noticed in the anatomy of the heart. We now proceed to a more interesting portion of the work.

### Pathological Effects.

"Before entering into the description and theory of symptoms, it may be as well to notice some of the *morbid changes of structure* in the various parts of the heart, which are often found in connexion with congenital malformations; and some circumstances in the history of these changes, which have led to doubts as to the period of their formation. In the nineteen cases collected by M. Louis,* there were ten instances of diminution of the orifice of the pulmonary artery, while it occurred only once in the aorta. Disease of the tricuspid valve is more common than of the mitral; but both are rare. In the cases already quoted, dilatation of the right auricle was observed in 18; five times with hypertrophy, and twice with atrophy of its parietes. The right ventricle was dilated nine times; and in four of these it was also hypertrophied. Simple hypertrophy of the walls of this cavity occurred in six others. On the left side, dilatation of the auricle was seen eight times; of the ventricle, four. Hypertrophy of the former is mentioned in three instances; of the latter, in two only. It must strike every one that these appearances, as hypertrophy, retraction of the orifices by deposition or union of the valves, &c. are so analogous to other independent morbid states of the heart, that there can be little doubt that they must arise from similar causes, and are frequently formed long after birth. They have, however, in other cases been found at so early an age, as to be attributable only to a morbid action pre-

* "Observations suivies de quelques Considérations sur la communication des cavités droites avec les cavités gauches du cœur. Par M. Louis, Arch. Gén. de Med. Tome iii."

vailing during the period of fœtal development. To the latter must we refer many of those instances terminating fatally soon after birth; to the former, those which show themselves only at a later period.

This leads me to one of the most extraordinary circumstances in the history of the disease under consideration. Many of the subjects of malformation of the heart have lived for sixteen, twenty, or many more years, without betraying any striking symptom of the presence of such affection; and this has led to a doubt, whether the malformation has existed previously to the occurrence of the train of symptoms by which it is usually known. Most of the cases in which such a doubt is admissible, will be found to consist of simple communication of the cavities, by perforation of their *septa;* and in these instances, we think, the causes of this apparent anomaly may be made sufficiently obvious.

Laennec* conceives that the narrow oblique opening which is often seen between the plates of the valve of the *foramen ovale,* though preventing any admixture of the venous and arterial blood, is often the cause of a more serious affection; since, by a violent blow or sudden effort, it may easily become dilated, and then progressively enlarged. A case related by Corvisart† is generally cited, as most strongly confirmative of this opinion. A postillion, aged 57, who is not said to have shown any previous indications of diseased heart, was attacked with dyspnœa, palpitation, &c. after a severe injury from a carriage passing over him, and a violent blow in the epigastrium. He continued for three years to present, with alternate accessions and remissions, the same symptoms; and, after death, exhibited a *foramen ovale* an inch in diameter; dilatation of the right auricle, with dilatation and hypertrophy of the right ventricle. In other cases, the symptoms have first showed themselves after violent fits of coughing; as in *pertussis,* or other pulmonary affections.

The following considerations induce us to believe, that such causes can operate but very seldom, if ever. Cases are known in which the *foramen ovale* has been pervious, or the *septum ventriculorum* imperfect, though no indication of such formation was ever present during life, and the fact itself only discovered after death. The absence of symptoms, therefore, does not entitle us to conclude that congenital malformation may not exist.‡ It is not difficult to account for this seeming anomaly. As long as the circulation remains tranquil—the lungs continue healthy—a free passage exists through the pulmonary artery—and the two sides of the heart maintain their natural proportions;—even though there is an anormal communication between the two cavities, little blood will pass through it. For we must remember, that the fibres of the heart contract in such a direction as to urge the current of the blood rather in its natural course than to any other channel; and, also, that an equal power (relative to the force required) is exercised on either side, and, therefore, the inclination of the fluid to pass from one side to the

---

* Laennec on Diseases of the Chest, &c. Translated by Forbes, 1829, p. 630.

† Corvisart, Op. Cit. p. 279.

‡ "Of the former case, I have myself lately seen an instance in the Royal Infirmary of Edinburgh, the subject of which died of icterus. The heart was otherwise quite healthy."

other, is equally opposed. But only let this balance of the circulation be destroyed; let more blood be sent to the right side of the heart by emotion, or exercise; or from disease let congestion take place in the lungs; and the accumulated mass of blood, stimulating the heart to increased action, is driven to seek an outlet where it may, and at once passes through these anormal openings.

From the augmentation of the *mass* of the fluid, the cavities, especially the auricular, soon become dilated; while by the *increased action*, according to a general law in the development of muscles, increased growth or hypertrophy* is produced.

In opposition to the opinion maintained by some, that the communications between the ventricles are the effect of ulceration or rupture, we may urge, that the edges of the openings are mostly smooth and tendinous, not presenting the thick and rugged surface of ulceration, nor the ragged fibrous appearance of rupture; that they are not preceded by the inflammation, which must be the forerunner of an ulcer, nor is the death so sudden as it would be, if the heart was ruptured.† If we observe, too, the situations in which the openings generally occur, we shall find them at the upper and posterior parts of the interventricular septum, parts which, from their being last formed, are known to be most subject to retardment of development, but which are least likely, for the occurrence of ulceration or rupture.‡ If to these it be added, that in cases which from birth have shown indications of the disease, we find exactly the same appearances, as in those apparently affected only a few months before death, we shall have, I think, sufficient reason to conclude, that these malformations are almost always of congenital origin." 44.

### Symptomatology.

This may be considered in relation to the functions of circulation, respiration, and nutrition. Auscultation has been but very seldom applied to the recognition of cardiac malformation; but it is very probable that the stethoscope will prove a powerful auxiliary in this department of investigation. The congenital contraction of the orifices is indicated by the *bruit de soufflet*.

"The dilatation and hypertrophy of the right side, the symptoms of which are pretty well established, are much more rare than on the left, except when combined with malformations, and will therefore of course help us in our diagnosis. Frequent palpitations on slight exertion, is a symptom very

* "The opinion of Corvisart, that the hypertrophy of the right side depends on the entrance of arterial blood into its cavities, and was caused by its more stimulating qualities, is not tenable. Some would be inclined to attribute it to the effect of inflammation, but we do not see why the heart should not follow the same laws of development as the voluntary muscles; nor do we think there are any facts which contradict it."

† Bertin, Traité des Maladies du Cœur, &c. p. 395.

‡ Bertin, p. 394, and Andral, Anat. Pathol. T. ii. p. 307.

common in these affections, and on careful inquiry is generally found to have existed from birth, though perhaps little noticed. When the disease has fairly established itself by the morbid formations already described, the patient is liable to severe paroxysms of dyspnœa, during which, palpitations are very apt to occur, though the heart's action may be sufficiently regular at other times. It is subject, however, to great variety in this respect. In one we find 'the action of the heart strong, but felt only at intervals,'* in another so strong as to be seen at a distance,† while in a third it is so weak as scarcely to be perceived. Corvisart‡ in one instance, mentions that 'la main, placée sur la région du cœur, sentait un battement peu régulier, accompagné d'un *bruissement* particulier trés-rémarquable.' There was here absence of one of the aortic valves, on which this phenomenon probably depended.

The pulse is scarcely more certain in its indications. When the body is at rest, the pulse is frequently quite natural. During the paroxysm, it is in some intermittent, in others regular; in some strong and full, and again in others almost imperceptible. It is not always synchronous with the heart;§ perhaps irregularity is its most general character. It should be understood, that we do not consider these changes in the circulation to depend on the original malformation, but to arise rather from the subsequent disease of other parts of the heart.

From the congestion, which we have already stated to be one of the effects of these malformations in their later stages, hemorrhages are of frequent occurrence. These mostly proceed from the nose or gums, sometimes from the stomach, intestines, or lungs. It has been noticed in the lungs particularly, when combined with tubercles there.|| These hemorrhages are generally trifling, and not as some have supposed, the immediate cause of death in most instances.

The changes in the state of the brain and mental functions, probably dependent on some alteration in the cerebral circulation, are headache, vertigo, and excessive irritability of temper. Farre¶ includes also, torpor of the brain, epilepsy, apoplexy, paralysis, and syncope. Of these latter, the first may be seen in cases of partial idiocy, but would rather come under the head of nutrition; the three next do not appear to be of frequent occurrence, and are rather coincidences than effects, while the last we are inclined to consider misnamed. Instead of syncope, dependent on an imperfect supply of blood by the arteries, and attended by paleness of the surface, want of action in the heart, &c. &c. it is probably asphyxia, arising, according to Bichat, from the transmission of black blood through the brain. It is certainly attended with most of the appearances usually seen in such cases.

The torpor of the brain is sometimes observed in common with that of other parts of the body, and seems to owe its origin to the defective stimulus afforded by the black blood with which the arteries are filled.

---

* Dr. Abercromby's Contributions to the Pathology of the Heart, Edin. Med. Chirurg. Trans. Vol. i. p. 59.

† Dr. Hunter, Med. Observ. Vol. vi.

‡ Corvisart, Op. Cit. p. 276.

§ Corvisart, Op. Cit. p. 277.

|| Louis, Arch. Gén. de Méd. Tome iii. p. 331, and Farre, p. 34.

¶ Farre, Op. Cit. p. 33.

Irritability of temper is quoted by Corvisart* in a case from M. Caillot, among others, and appeared to have been present from birth. It is often very strongly marked, and frequently induces the recurrence of the paroxysm.

The palpitations and disturbance to the circulation, in many instances, seem to be influenced by the state of the stomach. In some, the simple distention of the part will explain the impediment offered to the heart's action, but in others, according to Nasse,† it arises from indigestion, and is variously affected according to the nature of the food taken.

The pulsation of the jugulars, though noticed but rarely, is probably a very common symptom, from the frequent occurrence of dilatation of the right auricle." 47.

*Perspiration*. This is the function which suffers most in this unfortunate class of diseases. The breathing is habitually short and difficult—easily excited by slight exertions, or violent mental emotions—and often terminating in dreadful paroxysms of dyspnœa. The sense of suffocation is then intense—the body is more or less convulsed—the face assumes a dark purple colour, and becomes colder than natural—the eyes are prominent—the face bloated—the jugulars turgid—the respiration deep, with occasional screaming—the decubitus forward or abdominal—death.

"The paroxysms‡ sometimes occur periodically, and are frequently the immediate cause of death. Their duration is very various, from a few minutes to several hours. Patients generally recover from this state with a sigh or yawn, and even when it has been most severe, express themselves much more relieved than before its occurrence.§ How is this symptom to be explained? Mr. C. Bell || has supposed, that, as during the occurrence of the fit, the patient either leans forward, lies on the belly, presses the breast against something hard, or supports the abdomen with the hands; and as, from assuming the decumbent posture, the fit has been put off; ¶ therefore, this state depends on a want of pure air in the lungs, and this effort is made to contract the cavity of the chest, and expel as much as possible of the impure air. When this is as far as possible accomplished, continues the same author, the sigh or yawn, by which the recovery is preceded, again fills the lungs, and the patient finds himself suddenly relieved. But it is not in the lungs the disturbance first commences, and though first manifested

---

* Corvisart, Op. Cit. p. 294.

† Reil's Arch. für die Physiol. Vol. x. p. 276.

‡ Duncan's Medical Commentaries.

§ Corvisart, Op. Cit. p. 277.

|| Anatomy and Physiology of the Human Body. (6th Ed.) Vol. ii. p. 72.

¶ 'Any hurry upon his spirits, or brisk motion of his, would generally occasion a fit. And for some of the last years of his life, he has found out, by his own observation, that when the fit was coming upon him he could escape it altogether, or at least take considerably from its violence or duration, by instantly lying down upon the carpet on his left side, and remaining immoveably in that position for about ten minutes. I saw the experiment made with success.'—Dr. Hunter in Med. Observ. and Enquiries, Vol. vi. p. 301.

by this organ, it is probably only from sympathy with the brain it is affected. The causes usually exciting the paroxysm, are such as increase the rapidity of the venous circulation, and consequently overload the right side of the heart with blood; which in these malformed hearts is immediately transmitted unchanged to the left side, through the anormal communication. Hence, the blood passes forward to the brain, and produces complete or partial asphyxia; till at last the circulation again becomes equalized, and a sufficient quantity of oxygenized blood gains the left side, (for some portion still continues to pass through the lungs,) when the organs again receiving their proper stimulus, again resume their natural functions. The peculiar posture is chosen probably, as the upright in ordinary cases of dyspnœa, to allow the more easy passage of the blood from the lungs to the left side of the heart, whereby the former organ is relieved; while the fits have been put off or mitigated by lying on the belly or side, merely, as we believe, because in that position the body is most completely at rest, and the circulation becomes soonest tranquillized." 48.

Two other morbid effects arise from this mixture of black and red blood—the morbus ceruleus, and an unnaturally low temperature of the surface. Physiologists are divided about the cause of the blue skin in malformations of the heart. Some affirm that it is owing to the circulation of black or venous blood in the arteries—others, that it results from venous congestion in the capillaries. The arguments on both sides are well handled by our author, and are interesting in themselves.

"It is argued, that CYANIA does not depend on the circulation of black blood, because this symptom is not present in all cases of anormal communication; because it is not peculiar to these cases; because the blue colour is not general; because it is never present, except where there is contraction of the orifices causing congestion;—and lastly, because the colour of the fœtus at the birth is not purple.

We have already shown, that these malformations may exist from birth, without allowing this mixture of the two sorts of blood; and therefore it is no argument, that *cyania* does not always accompany them.

That the purple colour of the skin is not peculiar to such affections, is true only to a certain extent. In dilatation of the cavities of the right side of the heart,—in contraction of the orifices of that part,—in hepatization or emphysema of the lungs,—in spasmodic asthma,—or in hooping-cough,—in short, in all diseases impeding the circulation, and respiration, the skin becomes of a darker colour than natural. But it is on the extent to which this prevails, that the importance of the appearance as a diagnostic in this disease depends. The colour is in general much darker than in any simple congestion;* much more universal, much more constant; and frequently

* "I know of only two cases, which seem to contradict this. They are recorded by Dr. Thomson in the 12th Vol. of the Ed. Med. and Surg. Journal. In both the blueness is said to have been general, and in both it apparently arose from suppression of the catamenia, after exposure to cold."

extending to the brain and other viscera. This statement is borne out by the very first case, quoted by M. Louis* himself, and still more so, by another from Caillot;† 'les téguments de la face, de la poitrine, et des membres, étaient d'un violet tirant sur le noir; les intestins et les autres viscères abdominaux d'un brun foncé. A peine pouvait on distinguer dans le cerveau le substance corticale de la médullaire.'

The statement, that cyania is only present where there is contraction of the orifices, or dilatation, producing congestion, is contradicted by the case of transposition of the aorta and pulmonary artery already cited from Baillie,‡ where the heart 'had nothing else remarkable in its structure,' but where 'the child had a most unusually livid skin.'

The last argument originating with M. Fouquier, that the fœtus which circulates only black blood has not a blue skin, requires some consideration. In the *first* place, the colour of the fœtus at birth, and for some days after, is darker than at a later period;§ and, 2*d*, though it has been stated by Bichat,|| and others after him, that the colour of the blood in the umbilical arteries and veins is uniformly dark, yet we are justified in disputing this opinion, great as is the authority, as well from reasons drawn from analogy, as from the positive experience of other authors. In alluding to this subject, Bostock¶ observes, 'I cannot but feel surprise at such an opinion, as in some cases where I have had an oppotunity of examining the fœtus immediately after its extraction from the uterus, the different colours of the blood in the funis appeared quite obvious, thus agreeing with the observations of Dr. Jeffray, De Placenta, p. 41.' To this we may add the still more recent authority of Dr. Holland.** 'From the kindness of my friend Mr. Carr, surgeon, Sheffield, I have been enabled to prove, by an experiment of the simplest kind, that the umbilical vein circulates arterial blood.'—'But on taking part of the cord, as soon as the child was born, around which I had previously tied a ligature, about two or three inches from the free extremity, and cutting this with a sharp scalpel, in order to make an even surface, I very clearly discerned, on pressing the cord from below upwards, blood of a very different colour flowing from the umbilical veins and arteries.'

That cyania does depend, then, principally on the circulation of black blood in the arteries we maintain, because, as the colour of any organ is partly derived from the blood it contains, a change in the colour of the

---

* Arch. Gén. de Méd. t. iii. p. 326.

† Bulletin de la Faculté de Med. Année 1807, p. 21.

‡ Baillie's Morbid Anat. p. 36.

§ "I have more than once observed, in attending deliveries many years ago, that the fœtus had, at first exclusion, a dull dingy colour, but the moment it began to cry lustily, it immediately assumed a vivid red colour."—Ed. of Edin. Med. and Surg. Journ.

|| Bichat, Anat. Gén. 1801, t. ii. p. 343; and Magendie's Comp. of Physiol. by Milligan, p. 505, &c. &c.

¶ Bostock's Elementary System of Physiology, vol. ii. p. 199, note.

** The Physiology of the Fœtus, Liver, and Spleen. By George Calvert Holland, M.D. London, 1831, p. 154.

blood must affect the colour of the organ; and because, in these cases, the colour is deeper, more diffused, and more constant than in any other.*

In most of the cases recorded with any degree of care or minuteness, a great sensibility to cold, as well as a diminished temperature of the extremities or surface generally, has been particularly noticed. This symptom is aggravated by cold weather, and during the paroxysm of dyspnœa, while it is relieved by rest, coverings of flannel and the warm bath. Nasse and Farre seem to have been the first who thought of ascertaining by the thermometer to what extent this variation proceeded, and in the small number of cases in which it was tried, were surprised to find the temperature of the internal parts maintaining its usual elevation, while on the external it varied considerably, as much as from 74° to 98° Fahr. It would, therefore, appear, that it was rather a want of power to resist cold, than any diminution in the evolution of heat, on which this symptom depended. We have placed this under the head of lesion of the respiratory function, because it seems pretty certain that the evolution of animal heat is intimately connected with the changes which the blood undergoes in respiration, however difficult or inexplicable this process may be." 52.

The defect of nutrition which we generally observe in malformations of the heart, is probably owing to the anormal state of the blood itself, rendered unfit for its nutritious office.

We need not dwell on the subject of diagnosis, after the long catalogue of symptoms which have been given. Although these symptoms cannot all be expected in any one case, yet several of them are sure to be found in each.

### TREATMENT.

"The treatment of these affections may be discussed in a few words; it can only be palliative. The chief indications appear to be, to remove any cause exciting the recurrence of a paroxysm, and to relieve it when present. The first indication is best fulfilled by absolute rest; low diet, but such as contains most nutriment in a small compass, that the stomach may not be distended; absence of any excitement to the feelings or passions; diaphoretics; and the enjoyment of a moderately warm, regulated temperature. The last, by the abdominal decubitus, frictions with flannel, or, still better, the warm bath. To speak of the use of opiates, purgatives, and, in the last stage, diuretics, is unnecessary. Farre† has ingeniously supposed, that the skin in these cases may, to a certain extent, compensate for the imperfect manner in which the respiratory function is performed; and it is by acting on this surface, he is inclined to think, we must expect to afford the greatest

* "There are three cases on record, (vid. Corvisart, p. 296, Arch. Gén. t. iii. p. 336, and Farre, p. 15) which are exceptions to this remark, for, in these, either the skin was constantly pale, or it was sometimes pale and sometimes purple during the paroxysm. In the first of these cases, the orifice of the pulmonary artery was very much contracted, and it is therefore equally inexplicable on any other supposition hitherto adduced."

† Farre, Op. cit. p. 45.

relief. From the well known sympathy between the liver and lungs, and from the capability of the former organ to take on vicariously the function of the latter, as established by Tiedeman, it is not impossible, that, by stimulating this viscus to increased action, some of the bad effects, resulting from mal-oxygenation of the blood might be prevented." 54.

We trust that this thesis is only an *avant-courier* of some other and more extended performance. It augurs well for the future *practitioner*, when we find that the *student* has excelled in elementary knowledge and literary research. Judging from the specimen now before us, we would say that Dr. Paget bids fair to distinguish himself as a practical physician, a discerning physiologist, and an elegant writer. *Perge.*

